# Study Design and Participants’ Profile in the Sub-Cohort Study in the Japan Environment and Children’s Study (JECS)

**DOI:** 10.2188/jea.JE20200448

**Published:** 2022-05-05

**Authors:** Makiko Sekiyama, Shin Yamazaki, Takehiro Michikawa, Shoji F. Nakayama, Hiroshi Nitta, Yu Taniguchi, Eiko Suda, Tomohiko Isobe, Yayoi Kobayashi, Miyuki Iwai-Shimada, Masaji Ono, Kenji Tamura, Junzo Yonemoto, Toshihiro Kawamoto, Michihiro Kamijima, the Japan Environment and Children’s Study Group

**Affiliations:** 1 Japan Environment and Children’s Study Programme Office, National Institute for Environmental Studies, Ibaraki, Japan; 2 Department of Environmental and Occupational Health, School of Medicine, Toho University, Tokyo, Japan; 3 Department of Environmental Health, University of Occupational and Environmental Health, Fukuoka, Japan; 4 Department of Occupational and Environmental Health, Nagoya City University Graduate School of Medical Sciences, Nagoya, Japan

**Keywords:** birth cohort, profile, environmental exposure

## Abstract

**Background:**

The Japan Environment and Children’s Study (JECS) is a nationwide birth cohort study investigating environmental effects on children’s health and development. A Sub-Cohort Study has begun, conducting extended exposure and outcome measurements by targeting a subgroup randomly selected from the JECS Main Study. We report the Sub-Cohort Study methodology and participants’ baseline profiles.

**Methods:**

Of 100,148 children in the JECS Main Study, children born after April 1, 2013 who met eligibility criteria ([1] all questionnaire and medical record data from children and their mothers collected from the first trimester to 6 months of age, [2] biospecimens [except umbilical cord blood] from children and their mothers collected at first to second/third trimester and delivery) were randomly selected for each Regional Centre at regular intervals. Face-to-face assessment of neuropsychiatric development, body measurement, paediatrician’s examination, blood/urine collection for clinical testing and chemical analysis, and home visits (ambient and indoor air measurement and dust collection) are conducted. Participants are followed up at 1.5 and 3 years old for home visits, and 2, 4, 6, and 8 years old for developmental/medical examination. The details of protocols after age 10 are under discussion.

**Results:**

Of 10,302 selected children, 5,017 participated. The profiles of the participating mothers, fathers and children did not substantially differ between the Main Study and Sub-Cohort Study.

**Conclusion:**

The JECS Sub-Cohort Study offers a platform for investigating associations between environmental exposure and outcomes.

## INTRODUCTION

The Japan Environment and Children’s Study (JECS) is a nationwide birth cohort study designed to examine the effects of various environmental factors on children’s health and development.^1^^–^^4^ The main part of JECS (Main Study) aims to recruit 100,000 mother–child dyads, and to collect data from all participants. The study is designed to assess the effect of environmental factors, including exposure to chemical substances, on the proportion of reproduction/pregnancy complications, congenital anomalies, developmental disorders and immune/metabolic system dysfunction. Environmental exposure is assessed using self-administered questionnaires, by conducting numerical modelling and by biomonitoring methods applied to biological samples such as blood, urine, hair, breast milk and deciduous teeth.

For some exposure and outcome measurements, home visits and person-to-person examination are required. Such measurements include indoor air quality, house dust analysis, neurodevelopmental tests and paediatric examination. To examine the effects of environmental factors on developmental disorders (eg, attention deficit hyperactivity disorder [ADHD], which is one of the major target outcomes of the JECS), detailed on-site neurodevelopmental testing is necessary. Because of the practical issues and cost of sampling and analysis, however, these measurements cannot be applied to a sample as large as 100,000 participants. Thus, a Sub-Cohort Study was established to perform these extended exposure and outcome measurements for a subgroup randomly selected from the Main Study.

The Sub-Cohort Study is planned to conduct with 5,000 participants who are randomly selected from all of the participants in the Main Study. The participants are recruited from all of the Regional Centres involved in JECS. The Sub-Cohort Study employs face-to-face assessment of neuropsychiatric development, body measurement, paediatrician’s examination, blood/urine collection for clinical testing and chemical analysis, and home visits (ambient and indoor air measurement and dust collection).

In the current article, we first aimed to describe in detail the methodology used in the JECS Sub-Cohort Study. Second, we sought to compare the baseline profiles of participants in the Sub-Cohort Study with those in the Main Study reported in our previous paper.^2^

## METHODS

### Study participants

The details of study participants in the JECS Main Study have been described elsewhere.^2^ Briefly, from January 2011 to March 2014, women were recruited as early in pregnancy as possible at 15 Regional Centres throughout Japan, aiming to recruit 100,000 mother–child dyads. The respective distributions of maternal and infant characteristics in JECS were comparable to those obtained in the national survey.^5^

The total number of participants in the Sub-Cohort Study is planned to be 5,000. This number was determined to enable us to test hypotheses regarding to diseases with a relatively high prevalence (eg, obesity, allergic disease) with sufficient statistical power. For example, for obesity with an incidence rate of 10%, sample size of 5,000 provides sufficient power (>80%) to detect a hazard risk >2.0, in a case where the proportion of individuals exposed was 0.03. Similarly, for asthma (with an incidence rate of 2.4%) and ADHD (with an incidence rate of 3.0%), the corresponding power exceeded 80%, in cases where the proportion of individuals exposed was 0.1. The participants of the Sub-Cohort Study are prospective participants (children) of the Main Study who were born after April 1, 2013 and were permitted to continuously participate in the Sub-Cohort Study through written consent obtained from their mothers or legal guardians. The eligibility criteria for participants were as follows: (1) all of the questionnaire data and medical record transcripts of children and their mothers were collected from the first trimester to 6 months of age; and (2) biospecimens (except for umbilical cord blood) of children and their mothers were collected at the first trimester, second/third trimester, and delivery.

Among the participants of the Main Study meeting the criteria for being a participant of the Sub-Cohort Study, the Programme Office randomly selected children and created recruiting lists for each Regional Centre at a regular interval using a data management system, and distributed the list to each Regional Centre. The total number of children on the recruiting list is determined by taking account of the proportion of prospective participants who would be likely to give consent to participate at each Regional Centre, estimated based on the rates of recruitment of participants for the Main Study. The number of children selected for the list who reside in the Study Area of each Regional Centre is proportional to the number of the participants of the Main Study in the area. If a Regional Centre has several Study Areas that are geographically separated from each other, recruiting lists are created separately for each of the Study Areas.

Each Regional Centre sends a document including information about the Sub-Cohort Study to the parents of the children on the lists. The Regional Centre then contacts the participants’ mothers by telephone to confirm that they plan to continue to live in the Study Area until their children reach age 4. If the child and his/her parent (or a legal guardian) are considered to be unlikely to move out of the Study Area, the parent is provided with detailed information and asked to provide consent for their child’s participation in the Sub-Cohort Study. For each child on the recruiting list, the Regional Centre follows this procedure in order, until the number of the children reaches predetermined number. If the number of the children with an agreement to participate is not sufficient even after all of the children on the recruiting list have been contacted, the Programme Office repeats the sampling procedure to create an additional recruiting list.

At the initial home visit for environmental measurements (ie, on the first day of data collection for the Sub-Cohort Study), before data collection begins, the participant’s mother (or legal guardian) was asked to review information about the Sub-Cohort Study and sign the consent form.

### Assessments during pregnancy and at delivery

**Figure 1.**
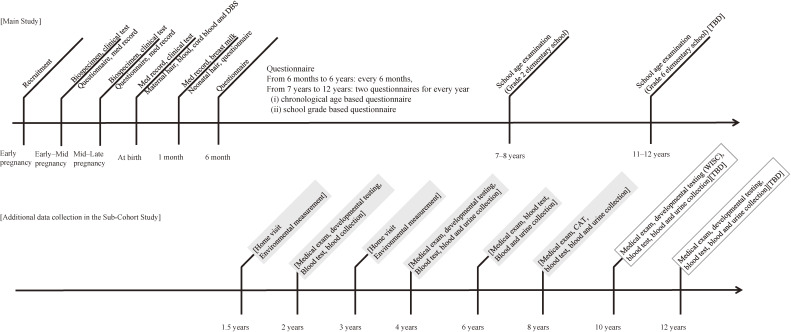
The JECS Sub-Cohort Study data collection timeline. DBS, dried blood spot; CAT, computer assisted tests; WISC, Wechsler Intelligence Scale for Children; TBD, to be determined.

As explained above, participants in the Sub-Cohort Study were also participants in the Main Study meeting the criteria for being a participant of the Sub-Cohort Study. Thus, participants completed the assessments of the Main Study during pregnancy and at delivery, which included questionnaires with medical record transcripts and collection of biospecimens (Figure 1).

#### Questionnaires

As previously described in detail,^2^ self-administered questionnaires were completed by pregnant women during the first trimester and second/third trimester to collect information on demographic factors, medical and obstetric history, physical and mental health, lifestyle, occupation, environmental exposure at home and in the workplace, housing conditions, and socio-economic status. Medical records were also transcribed by medical staff or a Research Co-ordinator.

#### Biospecimens

Biospecimens (blood, urine, hair, and umbilical cord blood) were collected during pregnancy and at delivery and were stored at −80°C in freezers, liquid nitrogen tanks, or at room temperature, while controlling temperature and humidity conditions.^2^

### Overall data collection schedule of the JECS Sub-Cohort Study

Figure 1 shows the overall data collection schedule of the JECS Sub-Cohort Study. Outcome variables are measured through developmental testing and medical examination when the participants reach 2 and 4 years of age, whereas environmental measurements are completed at 1.5 and 3 years, respectively, before the measurement of outcome variables. Subsequently, we collect health outcome information at 6, 8, 10, and 12 years of age. The environmental exposure data will be collected once or twice after the age of 10. The details of protocols at 10 and 12 years of age will be determined in accordance with the Main Study protocols, priority of outcome and exposure measurements, and budget. The protocol will finally be approved by the Steering Committee, then by the Institutional Review Boards (IRBs) of the participating institutions. If changes to the protocol are made, approval from IRBs will be obtained, and necessary procedures will be undertaken.

### Measurement of outcome variables

#### Developmental testing

To assess outcome variables in the neuropsychiatric development domain, developmental/cognitive tests were administered individually to the participants (eTable 1). For developmental testing at 2 and 4 years of age, the Kyoto Scale of Psychological Development^6^ was used, as it is the most widely used developmental measure in clinical settings in Japan. The personnel who carry out this test were trained and certified by the Programme Office. No test is performed at age 6. At age 8, cognitive functions are evaluated using a combination of computer assisted tests (CAT) including the Continuous Performance Test,^7^ Mental Number Line,^8^ Dimensional Change Card Sorting Test,^9^ and Finger Tapping Test. We plan to conduct the Wechsler Intelligence Scale for Children (WISC)^10^ at 10 years of age. At 12 years of age, appropriate examinations will be determined.

#### Medical examination

Medical examinations are conducted by paediatricians (eTable 2). The medical examinations consist of measurement of height (SECA 213 Stadiometer; SECA, Hamburg, Germany), weight (Tanita WB-260A Digital Body Scale; Tanita, Tokyo, Japan for age 2 and 4, Tanita MC780A Body Composition Analyser, Tanita, for age 6 and 8), abdominal circumference, body composition (Tanita MC780A Body Composition Analyser; Tanita), pulse, respiratory rate, blood pressure (Dura Shock DS66 Trigger Aneroid sphygmomanometer; Welch Allyn, Skaneateles Falls, NY, USA), arm span, visual examination of head and neck, chest, abdomen, back, and skin (using United Kingdom Working Party criteria)^11^; blood and urine collection. We plan to conduct nitric oxide measurement (NIOX VERO Airway Inflammation Monitor; Circassia AB, Circassia AB, Uppsala, Sweden) in children’s breath and to collect spirometry data (HI-302U Spirometer; Nihon Kohden, Tokyo, Japan) at 8 years of age.

For the blood collection, paediatricians and co-medical staff receive specific training to provide care for the children to relieve pain and distress before/during/after drawing blood.^12^ The total amount of blood collected from 2- and 4-year-olds is 4 mL. At the ages of 6 and 8, 10 mL of blood are collected. We also plan to collect 10 mL of blood at 10 and 12 years of age.

The variables specifically measured through medical examinations are 1) immune system disorders/allergies (non-specific Immunoglobulin E [IgE], specific IgE, Immunoglobulin G [IgG], Immunoglobulin A [IgA] of inhalant and food antigens, measles antibody), 2) metabolism/endocrine system (thyroid stimulating hormone [TSH], free thyroxine [fT4], 25[OH] Vitamin D, thyroxine [T4], triiodothyronine [T3], free triiodothyronine [fT3], insulin-like growth factor 1 [IGF-1], luteinizing hormone [LH], follicle-stimulating hormone [FSH], estradiol [E2], testosterone, dehydroepiandrosterone sulphate [DHEA-S]), and 3) chemical substances (eg, persistent organic pollutants). We plan to undertake several additional measurements for 2) the metabolism/endocrine system, at the age of 12: haemoglobin A1c (HbA1c), glucose, insulin, low-density lipoprotein (LDL), high-density lipoprotein (HDL), triglycerides (TG) (eTable 3).

### Evaluation of environmental exposure at ages 1.5 and 3 years

Children’s environmental exposures are measured using ambient environmental measurements and biomonitoring. Environmental measurements include indoor and outdoor gaseous atmospheric contaminants by passive diffusion samplers, particulate matter (PM) by gravimetric determination and house dust collection. Dwelling observation is also conducted to observe possible environmental hazards inside and around the participants’ houses. These measurements are all performed by trained and certified field staff at each Regional Centre. Biospecimens collected during the medical examination are tested for clinical biochemistry and aliquoted into cryovials and stored at −80°C for subsequent chemical analysis.

#### Mite allergen/endotoxin in house dust

Mites and endotoxin are measured in dust collected from the mattress/futon on which the children regularly sleep. For this test, field staff will vacuum a specific area (50 cm × 1 m) of the children’s mattress/futon using a specified handy-vacuum cleaner with a designated filter for 2 minutes. Mite allergens were determined using Enzyme-Linked Immuno Sorbent Assay (ELISA). ELISA Kits (Der p 1 and Der f 1) were obtained from Indoor Biotechnologies Ltd. (Charlottesville, VA, USA). Endotoxin was measured using Kinetic Chromogenic LAL Assay (Kinetic-QCL) obtained from Lonza Japan (Tokyo, Japan).

#### Heavy metals/non-volatile organic compounds in house dust

House dust samples are collected using household vacuum cleaners. Participants are asked to install a specified vacuum cleaner bag provided by JECS and to collect dust in their usual manner for 1 month. Those who use vacuum cleaners without bags (eg, cyclonic vacuum cleaners) are asked to collect dust for 1 month and transfer the dust into a plastic bag provided by the field staff. House dust samples are sieved with 250-µm stainless-steel mesh and stored at −25°C for further chemical analysis such as heavy metals, perfluorinated alkyl substances, chlorinated persistent organic pollutants, brominated/phosphorus flame retardants, bisphenols, phthalates, and other substances.

#### Ambient and indoor gaseous atmospheric contaminants

Gaseous atmospheric contaminants are sampled using two pairs of three different passive diffusion samplers that are deployed in the room in which children spend most of their time (eg, living room or bedroom) and outside the house for approximately 7 days. Target compounds are formaldehyde, acetaldehyde, toluene, ethylbenzene, *m*,*p*- and *o*-xylene, styrene, *p*-dichlorobenzene, nitrogen dioxides (NO_2_), sulphur dioxides (SO_2_) and ozone (O_3_), which are analysed according to previous reports.^13^^–^^15^ Briefly, formaldehyde, acetaldehyde and ozone are adsorbed onto a DSD-BPE/DNPH sampler (Sigma-Aldrich, Tokyo, Japan) and analysed using a high-performance liquid chromatograph (Shimadzu, Kyoto, Japan) coupled to a photodiode array detector (Shimadzu) with an Ascentis RP-Amide (4.6 mm i.d. × 150 mm, 3 µm) as an analytical column. NO_2_ and SO_2_ are adsorbed onto a DSD TEA sampler (Sigma-Aldrich) and analysed using an ion chromatography coupled to an electric conductivity detector (Thermo Fisher, Tokyo, Japan) with an Ion Pac AS12A (4 mm i.d. × 200 mm) as an analytical column. Toluene, ethylbenzene, *m*,*p*- and *o*-xylene, styrene and *p*-dichlorobenzene are adsorbed onto a passive sampler with carbon beads (Shibata Scientific Technology, Tokyo, Japan) and analysed using a head-space gas chromatography coupled to a mass spectrometer (Agilent Technologies, Tokyo, Japan) with an InertCap AQUATIC-2 (0.25 mm i.d., 60 m, 1.4 µm film thickness) as an analytical column.

#### Ambient and indoor particulate matter

PM is measured using a gravimetric method modified from a previously published method.^16^ Briefly, PM samples are collected on a glass fibre filter (Pallflex TX40H120; Pall Corp, Port Washington, NY, USA) using a portable air pump (MP-Σ300NIIT; Shibata Scientific Technology) deployed at the same locations at which diffusion samplers are located (both indoors and outdoors). Filters are weighed before and after sampling and PM2.5 and PM10-2.5 are measured separately.

#### Dwelling observation

Room temperature and humidity are recorded during sampling of gaseous atmospheric contaminants. The field staff conduct observations using the dwelling observation sheet. They also fill out a sheet to collect information about commercial products (eg, insecticide and air fresheners) and the type and amount of chemical substances used in daily life.

### Evaluation of environmental exposure after age 4

Chemical substances to which children are being exposed on a daily basis may have relatively short biological half-lives (eg, phenols, pesticides, and phthalates). Because those compounds or their metabolites are often detected in urine, urine samples are collected from children at 4, 6, and 8 years of age and subjected to biomonitoring. We also plan to collect urine samples at 10 and 12 years of age. Blood samples will be analysed for persistent organic pollutants, chemicals with relatively long biological half-lives, toxic elements and essential elements.

#### Personal monitoring of gaseous atmospheric contaminants

Methodology using passive samplers or a gas sensor for personal monitoring of gaseous atmospheric contaminants and aldehydes is being developed. Appropriate modifications will be made according to the results of the methodological development.

### Ethical issues

The present study was conducted according to the guidelines specified in the Declaration of Helsinki, and all procedures involving human participants were approved by the Japan Ministry of the Environment’s Institutional Review Board on Epidemiological Studies (No. 100406001) and the IRBs of all participating institutions. Written informed consent was obtained from all participating mothers and fathers.

### Data analysis

In the current article, we summarised the profiles of mothers, fathers and children following the same procedure reported previously for the JECS Main Study.^2^ Briefly, mothers’ profile data were collected via questionnaire and medical record transcripts: age, marital status, family composition, and passive smoking from the first trimester questionnaire, educational background and household income from the second/third trimester questionnaire, smoking habits, alcohol consumption, and occupation in early pregnancy from the first trimester questionnaire supplemented with data from the second/third trimester questionnaire, pre-pregnancy height and weight and parity from the medical record transcripts. Profile data on male partners (fathers) via questionnaire: age and occupation during their partner’s early pregnancy, in addition to smoking habits, alcohol consumption, body mass index (BMI), and educational background. Profile data from medical record transcripts on the children were also summarised, including delivery information (live birth or not, singleton or multiple birth, gestational age at birth, sex, type of delivery) and anthropometry at birth. The present study is based on the dataset of jecs-ag-20160424, supplemented with some variables in the dataset of jecs-ta-20190930. Participants’ profiles were processed in aggregate and also separately for each of the 15 Regional Centres. Continuous data are described using arithmetic means, standard deviations (SD) and ranges; categorical and ordinary data by frequencies and percentages. All analyses were performed using SAS V.9.4 (SAS Institute, Inc, Cary, NC, USA) and SPSS 24.0 (IBM Corp, Armonk, NY, USA).

## RESULTS

**Figure 2.**
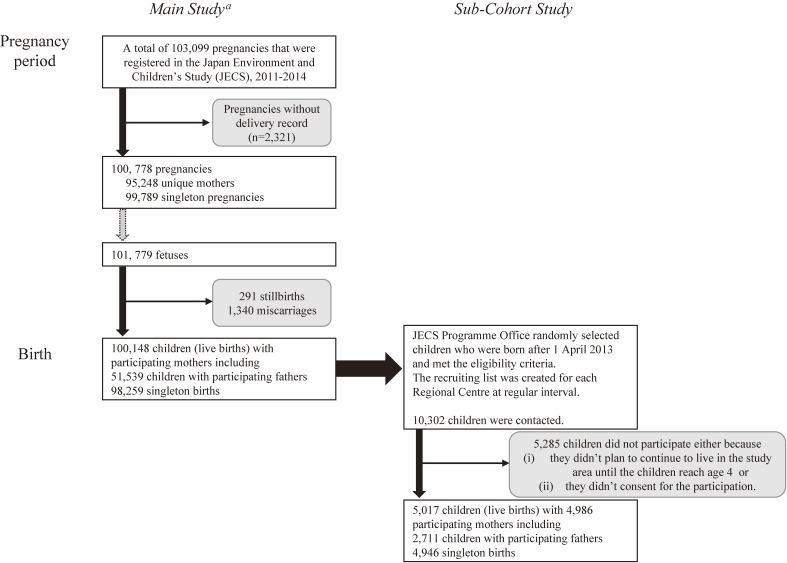
Flow chart of participants in the JECS Sub-Cohort Study. ^a^Michikawa et al, J Epidemiol 2018;28(2):99–104 (reference No. 2)

Figure 2 shows the flow chart of participants in JECS Sub-Cohort Study. First, 103,099 pregnancies were enrolled in the JECS Main Study. After excluding 2,321 pregnancies with no subsequent delivery record, there were 100,778 pregnancies resulting in delivery. These 100,778 pregnancies involved 101,779 foetuses and resulted in 100,148 live births, with 291 stillbirths (foetal deaths occurring at ≥22 weeks of gestation), and 1,340 miscarriages. Of 100,148 children (live births), the JECS Programme Office randomly selected children who were born after April 1, 2013 and met the eligibility criteria explained in the methods above. Recruiting lists were created for each Regional Centre at regular intervals, and a total of 10,302 children were contacted. Of 10,302 children, 5,285 did not participate, either because (i) they did not plan to continue to live in the Study Area until the children reached age 4, or, (ii) they did not consent to participation. The final analysis included 5,017 children, including 4,946 singleton births and 2,711 children with participating fathers.

Table 1 shows the number of mothers, fathers, and children who completed each survey item in the Sub-Cohort Study. Because the participants of the Sub-Cohort Study were selected from Main Study participants whose questionnaire (including medical record transcripts) and biospecimen (except for cord blood) data were collected from pregnancy to 6 months of age, the data were collected for almost all participants. Cord blood samples were collected from 4,573 of 5,017 children surveyed.

**Table 1.** Numbers of mothers, fathers, and children who completed each survey item in the JECS Sub-Cohort Study

	1^st^ trimester	2^nd^/3^rd^ trimester	Birth

	*n*	*n*	*n*
Mother (4,986 pregnancies)			
Questionnaire and interview about drug use	4,986	4,986	
Medical record transcription	4,986		4,986
Blood	4,686	4,958	4,986
Urine	4,656	4,950	
Hair			X^a^
Father (2,696 their partner’s pregnancies)			
Questionnaire^b^	2,672		
Blood^b^	2,652		
Child (*n* = 5,017)			
Medical record transcription			5,017
Cord blood			4,573

^a^We have not yet fixed the data.

^b^Between the mother’s early pregnancy and 1 month after delivery, we distributed a questionnaire to fathers, and collected blood from them.

Table 2, Table 3, and Table 4 show the baseline profiles of participants. Hereafter, we discuss the profiles following our previous paper.^2^ Baseline profiles of mothers in the JECS Main Study and Sub-Cohort Study are shown in Table 2. The mean age at delivery was 31.9 (SD, 4.9) years in the Sub-Cohort Study, whereas it was 31.2 (SD, 5.1) years in the Main Study. Most mothers were married (96.7% in the Sub-Cohort Study and 95.7% in the Main Study) and resided with their partner (and their child[ren]) (78.0% in the Sub-Cohort Study and 75.1% in the Main Study). The proportions of those who had received at least 13 years of education was 68.9% for mothers and 60.8% for fathers (mother-reported) in the Sub-Cohort Study, whereas it was 63.7% for mothers and 55.8% for fathers, respectively, in the Main Study. The distribution of household income peaked at 4 to <6 million Japanese-yen/year (34.3%) and 2 to <4 million yen/year (32.4%) in the Sub-Cohort Study, whereas it peaked at 2 to <4 million Japanese-yen/year (34.6%) and 4 to <6 million yen/year (33.1%) in the Main Study. The most common occupations for mothers in early pregnancy were homemaker (30.1%) and professional/engineering worker (24.4%) in the Sub-Cohort Study.

**Table 2.** Baseline profiles of mothers in the JECS Main Study and Sub-Cohort Study

Variables	JECS Sub-Cohort Study			JECS Main Study^a^		

	Number of valid response	*n*	(%)	Number of valid response	*n*	(%)
Number of pregnancies	4,986			100,778		
Age at delivery, years	4,985			100,768		
Total, mean (SD)		4,985	31.9 (4.9)		100,768	31.2 (5.1)
<20		17	0.3		893	0.9
20–24		339	6.8		9,229	9.2
25–29		1,241	24.9		27,686	27.5
30–34		1,791	35.9		35,571	35.3
35–39		1,295	26.0		22,713	22.5
≥40		302	6.1		4,676	4.6
Marital status	4,973			98,312		
Married		4,809	96.7		94,032	95.6
Unmarried		132	2.7		3,444	3.5
Divorced/widowed		32	0.6		836	0.9
Family composition	4,960			98,123		
One-person households		24	0.5		653	0.7
A couple only		1,567	31.6		30,105	30.7
A couple with their child(ren)		2,299	46.4		43,556	44.4
A parent with his or her child(ren)		25	0.5		848	0.9
Other households		1,045	21.1		22,961	23.4
Educational background, years	4,962			97,004		
<10		188	3.8		4,704	4.8
10–12		1,357	27.3		30,544	31.5
13–16		3,343	67.4		60,333	62.2
≥17		74	1.5		1,423	1.5
Paternal educational background, years	4,945			96,387		
<10		281	5.7		7,049	7.3
10–12		1,656	33.5		35,515	36.8
13–16		2,731	55.2		49,483	51.3
≥17		277	5.6		4,340	4.5
Household income, million Japanese-yen/year	4,762			90,596		
<2		221	4.6		5,140	5.7
2 to <4		1,544	32.4		31,311	34.6
4 to <6		1,634	34.3		29,942	33.1
6 to <8		786	16.5		14,410	15.9
8 to <10		357	7.5		5,926	6.5
≥10		220	4.6		3,867	4.3
Occupation in early pregnancy	4,948			97,935		
Administrative and managerial workers		25	0.5		567	0.6
Professional and engineering workers		1,208	24.4		21,857	22.3
Clerical workers		811	16.4		16,432	16.8
Sales workers		265	5.4		5,744	5.9
Service workers		732	14.8		15,527	15.9
Security workers		17	0.3		242	0.2
Agriculture, forestry and fishery workers		26	0.5		454	0.5
Manufacturing process workers		119	2.4		3,376	3.4
Transport and machine operation workers		9	0.2		177	0.2
Construction and mining workers		4	0.1		71	0.1
Carrying, cleaning, packaging, and related workers		41	0.8		678	0.7
Homemaker		1,490	30.1		28,225	28.8
Others (students, inoccupation, workers not classifiable by occupation)		201	4.1		4,585	4.7
Smoking habits	4,984			99,053		
Never smoked		3,068	61.6		57,444	58.0
Ex-smokers who quit before pregnancy		1,206	24.2		23,571	23.8
Smokers during early pregnancy		710	14.3		18,038	18.2
Passive smoking (presence of smokers at home)^b^	4,245			79,910		
No		3,697	87.1		66,486	83.2
Yes		548	12.9		13,424	16.8
Alcohol consumption	4,986			99,149		
Never drank		1,675	33.6		34,279	34.6
Ex-drinkers who quit before pregnancy		941	18.9		19,392	19.6
Drinkers during early pregnancy		2,370	47.5		45,478	45.9
Body mass index before pregnancy	4,985			100,538		
<18.5 kg/m^2^		794	15.9		16,272	16.2
18.5–24.9 kg/m^2^		3,657	73.4		73,416	73.0
≥25 kg/m^2^		534	10.7		10,850	10.8
Parity	4,968			100,288		
0		2,058	41.4		41,573	41.5
1		1,900	38.2		38,281	38.2
≥2		1,010	20.3		20,434	20.4

SD, standard deviation.

^a^Michikawa et al, J Epidemiol 2018;28(2):99–104 (reference No. 2).

^b^Excluding smokers during early pregnancy.

**Table 3.** Baseline profiles of fathers in the JECS Main Study and Sub-Cohort Study

Variables	JECS Sub-Cohort Study			JECS Main Study^a^		

	Number of valid response	*n*	(%)	Number of valid response	*n*	(%)
Number of their partner’s pregnancies	2,696			51,402		
Age when their children were born, years	2,696			51,104		
Total, mean (SD)		2,696	33.6 (6.0)		51,104	32.9 (5.9)
<20		2	0.1		198	0.4
20–24		122	4.5		3,173	6.2
25–29		581	21.6		11,659	22.8
30–34		857	31.8		16,867	33.0
35–39		708	26.3		12,738	24.9
≥40		426	15.8		6,469	12.7
Occupation during their partner’s early pregnancy	2,655			49,700		
Administrative and managerial workers		116	4.4		2,029	4.1
Professional and engineering workers		889	33.5		15,001	30.2
Clerical workers		253	9.5		4,627	9.3
Sales workers		263	9.9		5,366	10.8
Service workers		306	11.5		5,650	11.4
Security workers		116	4.4		2,065	4.2
Agriculture, forestry and fishery workers		53	2.0		929	1.9
Manufacturing process workers		319	12.0		6,744	13.6
Transport and machine operation workers		124	4.7		2,061	4.1
Construction and mining workers		146	5.5		3,413	6.9
Carrying, cleaning, packaging, and related workers		33	1.2		828	1.7
Homemaker		4	0.2		66	0.1
Others (students, inoccupation, workers not classifiable by occupation)		33	1.2		921	1.9
Smoking habits	2,664			49,815		
Never smoked		864	32.4		14,284	28.7
Ex-smokers who quit before their partner’s pregnancy		692	26.0		11,757	23.6
Smokers during their partner’s early pregnancy		1,108	41.6		23,774	47.7
Alcohol consumption	2,667			49,839		
Never drank		564	21.1		10,588	21.2
Ex-drinkers		101	3.8		1,873	3.8
Drinkers		2,002	75.1		37,378	75.0
Body mass index	2,650			49,532		
<18.5 kg/m^2^		93	3.5		1,797	3.6
18.5–24.9 kg/m^2^		1,822	68.8		34,204	69.1
≥25 kg/m^2^		735	27.7		13,531	27.3

SD, standard deviation.

^a^Michikawa et al, J Epidemiol 2018;28(2):99–104 (reference No. 2).

**Table 4.** Baseline profiles of children in the JECS Main Study and Sub-Cohort Study

Variables	JECS Sub-Cohort Study			JECS Main Study^a^		

	Number of valid response	*n*		Number of valid response	*n*	
Number of live births	5,017			100,148		
Singleton births, %		4,946	98.6		98,259	98.1
Gestational age at birth, weeks	5,017			100,148		
Total, mean (SD)		5,017	39.3 (1.5)		100,148	39.2 (1.7)
Preterm births (<37), %		226	4.5		5,599	5.6
Term births (37–41), %		4,783	95.3		94,322	94.2
Postterm births (≥42), %		8	0.2		227	0.2
Sex	5,017			100,137		
Male, %		2,554	50.9		51,316	51.2
Female, %		2,463	49.1		48,821	48.8
Type of delivery	5,009			99,884		
Vaginal, %		4,068	81.2		79,783	79.9
Caesarean, %		941	18.8		20,101	20.1
Birth weight, g	5,017			100,071		
Total, mean (SD)		5,017	3,040 (414)		100,071	3,008 (434)
Singleton births	4,946			98,182		
Total, mean (SD)		4,946	3,050 (405)		98,182	3,023 (420)
Male, mean (SD)		2,523	3,095 (413)		50,312	3,065 (426)
Female, mean (SD)		2,423	3,003 (391)		47,863	2,979 (408)
Low birth weight (<2,500), %		358	7.2		7,981	8.1
Birth height, cm	5,000			99,785		
Total, mean (SD)		5,000	49.0 (2.2)		99,785	48.8 (2.4)
Singleton births	4,929			97,912		
Total, mean (SD)		4,929	49.0 (2.1)		97,912	48.9 (2.3)
Male, mean (SD)		2,512	49.3 (2.2)		50,166	49.2 (2.3)
Female, mean (SD)		2,417	48.8 (2.0)		47,740	48.6 (2.3)
Birth head circumference, cm	4,990			99,538		
Total, mean (SD)		4,990	33.2 (1.5)		99,538	33.2 (1.5)
Singleton births	4,921			97,692		
Total, mean (SD)		4,921	33.2 (1.5)		97,692	33.2 (1.5)
Male, mean (SD)		2,510	33.5 (1.5)		50,054	33.4 (1.5)
Female, mean (SD)		2,411	33.0 (1.4)		47,633	33.0 (1.5)
Birth chest circumference, cm	4,988			99,489		
Total, mean (SD)		4,988	31.8 (1.8)		99,489	31.7 (1.9)
Singleton births	4,919			97,653		
Total, mean (SD)		4,919	31.9 (1.7)		97,653	31.8 (1.8)
Male, mean (SD)		2,510	32.0 (1.8)		50,034	31.9 (1.9)
Female, mean (SD)		2,409	31.7 (1.7)		47,614	31.6 (1.8)

SD, standard deviation.

^a^Michikawa et al, J Epidemiol 2018;28(2):99–104 (reference No. 2).

Baseline profiles of the fathers are shown in Table 3. The mean age of fathers when their partners gave birth was 33.6 (SD, 6.0) years in the Sub-Cohort Study, whereas it was 32.9 (SD, 5.9) years in the Main Study, and 33.5% were engaged in professional/engineering work. The proportions of smoking during early pregnancy (or partner’s early pregnancy) were 14.3% and 41.6% for mothers and fathers in the Sub-Cohort Study, respectively, and 18.2% and 47.7% for mothers and fathers in the Main Study, respectively. The proportion of passive smoking of mothers (Table 2) was 12.9% in the Sub-Cohort Study, whereas it was 16.8% in the Main Study.

Table 4 shows baseline profiles of the participating children. The mean gestational age at birth was 39.3 (SD, 1.5) weeks in the Sub-Cohort Study, whereas it was 39.2 (SD, 1.7) weeks in the Main Study. The secondary sex ratio (male/female) was 1.04. Among singleton birth males in the Sub-Cohort Study, the mean anthropometric values at birth were: weight, 3,095 (SD, 413) g; height, 49.3 (SD, 2.2) cm; head circumference, 33.5 (SD, 1.5) cm; and chest circumference, 32.0 (SD, 1.8) cm. In contrast, in the Main Study, the mean anthropometric values at birth were: weight, 3,065 (SD, 426) g; height, 49.2 (SD, 2.3) cm; head circumference, 33.4 (SD, 1.5) cm; and chest circumference, 31.9 (SD, 1.9) cm. Among singleton birth females in the Sub-Cohort Study, the mean anthropometric values at birth were: weight, 3,003 (SD, 391) g; height, 48.8 (SD, 2.0) cm; head circumference, 33.0 (SD, 1.4) cm; and chest circumference, 31.7 (SD, 1.7) cm. In the Main Study, the mean anthropometric values at birth were: weight, 2,979 (SD, 408) g; height, 48.6 (SD, 2.3) cm; head circumference, 33.0 (SD, 1.5) cm; and chest circumference, 31.6 (SD, 1.8) cm.

The baseline profiles of the mothers, fathers, and children for each Regional Centre are shown in eTable 4, eTable 5, and eTable 6.

## DISCUSSION

The JECS Main Study has established one of the largest birth cohorts in the world, covering all regions in Japan.^1^^,^^2^ In such a large cohort, detailed exposure measurements and clinical examinations are difficult to apply to all participants. Thus, by targeting a subgroup of participants randomly selected from the Main Study, we established a Sub-Cohort Study for extended analysis. The current article outlines the protocol and baseline profiles of the participants in the JECS Sub-Cohort Study.

The baseline profiles of participating mothers, fathers and children did not substantially differ between the Main Study and the Sub-Cohort Study. It should be noted, however, that the peak household income in the Sub-Cohort Study (4 to <6 million Japanese-yen/year) was higher than that in the Main Study (2 to <4 million Japanese-yen/year). Similarly, the proportion of passive smoking among mothers was lower in the Sub-Cohort Study (12.9%) than that in the Main Study (16.8%). Other large-scale birth cohort studies, such as the Norwegian mother and child cohort study (MoBA) and the Danish national birth cohort study (DNBC), reported that participation in the Sub-Cohort Study was associated with participants’ socio-economic status and lifestyle factors.^17^^,^^18^ Although such a socio-economic gradient might influence some of the prevalence estimates, as reflected in the slightly lower proportions of low birth weight (<2,500 g) in the Sub-Cohort Study (7.2%) compared with the Main Study (8.1%), it would not be expected to influence the estimates of exposure-outcome associations, which was the main objective of the JECS Sub-Cohort Study.

The JECS Sub-Cohort Study has several important strengths that should be noted. First, the large amount of information collected via home visits and person-to-person examinations allowed us to investigate the associations between environmental exposure and outcomes in a setting where various social- or lifestyle-related covariates collected from questionnaires and analysis of biological samples can be controlled for. Second, JECS employs not only a cross-sectional approach, but also a longitudinal design, to examine the onset of diseases as well as changes in the severity of symptoms, scores of developmental tests and biomarkers, as results of exposure to environmental factors. In addition, by comparing the results of the Sub-Cohort Study and those of the Main Study in which most data are collected via a questionnaire, the validity of questionnaires is evaluated. Finally, compared with other epidemiological studies, the JECS Sub-Cohort Study has maintained a strikingly high participation rate because of the tremendous effort made by the 15 Regional Centres; of 5,017 participating children, 4,774 children have continuously been followed up as of October 26, 2020.

Information about the JECS is available to the public at https://www.env.go.jp/chemi/ceh/en/index.html. To date, we have published 130 journal papers (as of November 20, 2020) mainly using data collected in the Main Study. We will soon be able to report the results of the Sub-Cohort Study using various environmental exposure, developmental testing and medical examination data to examine the associations between exposure and health outcomes.

## APPENDIX A. SUPPLEMENTARY DATA

eTable 1. Developmental tests in the Sub-Cohort Study

eTable 2. Medical examination in the Sub-Cohort Study

eTable 3. Blood tests and biomonitoring items in the Sub-Cohort Study

eTable 4. Selected baseline profile of mothers according to Regional Centre in the JECS Sub-Cohort Study

eTable 5. Selected baseline profile of fathers according to Regional Centre in the JECS Sub-Cohort Study

eTable 6. Selected baseline profile of children according to Regional Centre in the JECS Sub-Cohort Study

## APPENDIX B.

Members of the JECS Group as of 2020: Michihiro Kamijima (principal investigator, Nagoya City University, Nagoya, Japan), Shin Yamazaki (National Institute for Environmental Studies, Tsukuba, Japan), Yukihiro Ohya (National Center for Child Health and Development, Tokyo, Japan), Reiko Kishi (Hokkaido University, Sapporo, Japan), Nobuo Yaegashi (Tohoku University, Sendai, Japan), Koichi Hashimoto (Fukushima Medical University, Fukushima, Japan), Chisato Mori (Chiba University, Chiba, Japan), Shuichi Ito (Yokohama City University, Yokohama, Japan), Zentaro Yamagata (University of Yamanashi, Chuo, Japan), Hidekuni Inadera (University of Toyama, Toyama, Japan), Takeo Nakayama (Kyoto University, Kyoto, Japan), Hiroyasu Iso (Osaka University, Suita, Japan), Masayuki Shima (Hyogo College of Medicine, Nishinomiya, Japan), Youichi Kurozawa (Tottori University, Yonago, Japan), Narufumi Suganuma (Kochi University, Nankoku, Japan), Koichi Kusuhara (University of Occupational and Environmental Health, Kitakyushu, Japan), and Takahiko Katoh (Kumamoto University, Kumamoto, Japan).
