# Supplementary material for: Study Design and Participants’ Profile in the Sub-Cohort Study in the Japan Environment and Children’s Study (JECS)
Source: J Epidemiol. 2022 May 5;32(5):228–36. doi: 10.2188/jea.JE20200448 (PMC8979916; doi:10.2188/jea.JE20200448)
Supplement: Supplementary file 1 [file je-32-228-s001.pdf]

eTable 1. Developmental tests in the Sub-Cohort Study

	2 years	4 years	6 years	8 years	10 years	12 years
Kyoto Scale of Psychological Development	X	X				
Wechsler Intelligence Scale for Children (WISC)-IV					TBD	
CAT (Continuous Performance Test, Mental Number Line, Dimensional Change Card Sorting Test, Finger Tapping Test)				X		TBD

TBD, to be determined; X, The item was measured.

eTable 2. Medical examination in the Sub-Cohort Study

	2 years	4 years	6 years	8 years	10 years	12 years
Height, weight	X	X	X	X	TBD	TBD
Abdominal circumference			X	X	TBD	TBD
Body composition			X	X	TBD	TBD
Vital sign (pulse, respiratory rate)	X					
Blood pressure	X	X	X	X	TBD	TBD
Arm span			X			TBD
Skin examination (UK Working Party criteria)	X	X	X	X	TBD	TBD
Exhaled NO, spirometry				X	TBD	TBD
Blood collection	X	X	X	X	TBD	TBD
Urine collection		X	X	X	TBD	TBD
Puberty					TBD	TBD
Microbiome					TBD	

TBD, to be determined; X, The item was measured.

eTable 3. Blood tests and biomonitoring items in the Sub-Cohort Study

	2 years	4 years	6 years	8 years	10 years	12 years
Non-specific Immunoglobulin E (IgE)	X	X	X	X	TBD	TBD
Specific IgE, Immunoglobulin A (IgA), Immunoglobulin G1 (IgG1) and Immunoglobulin G4 (IgG4)	X	X	X	X	TBD	TBD
Measles antibody				X		
Thyroid stimulating hormone (TSH), free thyroxine (fT4)	X	X	X	X	TBD	TBD
Thyroxine 4 (T4), triiodothyronine (T3), free triiodothyronine (fT3)				X	TBD	TBD
25(OH)Vitamin D	X	X	X	X	TBD	TBD
Insulin-like growth factor 1(IGF-1)			X	X	TBD	TBD
Luteinizing hormone (LH), Follicle-stimulating hormone (FSH), steroid hormones			X	X	TBD	TBD
Haemoglobin A1c (HbA1c), glucose, insulin, low-density lipoprotein (LDL), high-density lipoprotein (HDL), triglycerides (TG)						TBD
Chemical substances	X	X	X	X	TBD	TBD

TBD, to be determined; X, The item was measured.

eTable 4. Selected baseline profile of mothers according to Regional Centre in the JECS Sub-Cohort Study

Variables	Hokkaido (%)	Miyagi (%)	Fukushima (%)	Chiba (%)	Kanagawa (%)	Koshin (%)	Toyama (%)	Aichi (%)	Kyoto (%)	Osaka (%)	Hyogo (%)	Tottori (%)	Kochi (%)	Fukuoka (%)	South Kyushu / Okinawa (%)
Number of pregnancies (Main Study)	7,970	9,130	12,882	5,952	6,473	7,175	5,451	5,568	3,920	7,861	5,085	3,026	6,984	7,553	5,748
Number of pregnancies (Sub-Cohort Study)	404	447	632	299	324	357	270	278	192	386	246	146	339	386	280
Age at delivery, years															
Total, mean (SD)	31.8 (4.9)	31.0 (5.2)	31.3 (5.0)	32.3 (4.5)	32.6 (4.8)	32.4 (4.8)	32.5 (4.8)	32.2 (4.7)	33.0 (4.6)	31.8 (5.2)	32.2 (4.8)	31.8 (4.8)	32.0 (4.8)	32.4 (4.9)	31.2 (5.4)
<25	6.4	13.9	9.2	2.7	5.6	5.0	4.4	6.8	2.6	10.1	3.3	6.8	6.2	4.7	12.1
25–29	27.0	25.3	27.7	25.8	19.4	23.8	24.1	22.7	22.9	23.1	26.8	26.0	26.8	23.6	25.7
30–34	37.9	33.0	35.4	35.8	38.3	37.0	31.5	39.2	41.7	32.6	38.6	38.4	35.4	36.8	32.5
≥35	28.7	27.8	27.7	35.8	36.7	34.2	40.0	31.3	32.8	34.2	31.3	28.8	31.6	35.0	29.6
Marital status															
Married	95.5	95.0	95.7	97.7	96.9	97.5	97.8	98.2	99.5	96.4	98.0	97.3	95.6	97.4	96.0
Unmarried	3.5	4.1	3.8	1.7	2.8	2.0	2.2	1.4	0.0	2.8	0.8	2.1	3.6	1.8	3.6
Divorced/widowed	1.0	0.9	0.5	0.7	0.3	0.6	0.0	0.4	0.5	0.8	1.2	0.7	0.9	0.8	0.4
Family composition															
One-person households	1.7	0.4	0.6	0.0	0.0	0.8	0.4	0.0	0.0	0.8	0.4	0.0	0.9	0.0	0.0
A couple only	39.4	19.9	26.8	33.0	36.6	33.4	31.5	35.3	33.0	31.0	40.2	28.7	32.9	34.0	25.4
A couple with their child(ren)	46.9	36.5	39.7	46.5	47.8	43.5	41.6	48.2	51.8	52.1	51.2	38.5	52.5	54.8	49.8
A parent with his or her child(ren)	0.2	0.7	0.6	0.3	0.9	0.0	0.0	0.0	0.0	0.5	1.2	0.7	0.9	0.8	0.4
Other households	11.7	42.5	32.2	20.2	14.6	22.2	26.6	16.5	15.2	15.6	6.9	32.2	12.8	10.4	24.4
Educational background, years															
<10	3.2	6.5	3.0	2.0	2.2	2.0	2.6	3.6	1.6	6.8	6.1	4.1	3.5	3.9	4.6
10–12	31.1	39.5	35.5	25.3	20.5	25.0	20.5	23.0	12.6	23.2	24.4	29.5	20.1	23.9	39.3
13–16	63.9	53.4	61.0	71.3	75.8	71.3	74.6	70.5	83.2	68.0	66.7	64.4	76.1	70.4	55.7

≥17	1.7	0.7	0.5	1.4	1.6	1.7	2.2	2.9	2.6	2.1	2.8	2.1	0.3	1.8	0.4
Paternal educational background, years															
<10	3.8	7.4	4.7	4.1	5.9	3.7	4.5	7.9	2.1	8.6	4.9	8.9	7.1	5.7	6.4
10–12	32.8	49.0	42.7	31.8	23.8	30.4	32.2	18.1	23.0	34.6	29.3	34.2	30.1	29.6	41.4
13–16	58.3	41.3	49.1	56.1	64.1	60.6	54.3	65.0	63.4	51.8	59.8	51.4	58.0	59.2	48.2
≥17	5.3	2.3	3.5	8.1	6.3	5.4	9.0	9.0	11.5	5.0	6.1	5.5	4.8	5.5	3.9
Household income, million Japanese-yen/year															
<2	5.4	6.4	4.5	2.7	3.2	4.7	2.4	2.6	2.1	3.8	2.5	4.3	6.4	3.4	13.3
2 to <4	38.5	37.4	34.5	25.0	26.6	33.5	20.1	27.0	28.9	36.2	29.2	37.9	31.7	32.6	39.9
4 to <6	30.7	30.5	34.0	40.4	33.3	35.9	41.7	35.6	30.5	36.4	36.4	32.1	32.6	36.9	28.0
6 to <8	15.0	12.1	15.8	20.2	20.2	16.8	24.4	21.3	18.7	13.2	16.5	12.9	18.0	15.9	10.3
8 to <10	6.2	7.8	6.3	8.2	9.0	5.9	7.5	9.4	10.2	7.1	11.9	7.1	8.2	5.3	6.3
≥10	4.1	5.9	5.0	3.4	7.7	3.2	3.9	4.1	9.6	3.3	3.4	5.7	3.0	5.8	2.2
Occupation in early pregnancy															
Administrative and managerial workers	0.3	0.5	0.3	0.3	0.0	0.0	0.4	1.1	0.0	0.5	0.0	0.7	1.5	0.5	1.8
Professional and engineering workers	24.1	23.0	23.5	24.8	18.9	27.9	29.7	20.4	29.7	21.2	18.1	31.0	33.3	19.5	28.5
Clerical workers	13.9	14.9	17.8	12.4	16.2	16.7	19.0	18.6	15.1	17.2	14.0	17.9	18.5	13.5	21.3
Sales workers	5.2	5.0	5.4	5.0	7.5	6.9	4.5	7.6	4.2	3.4	3.7	3.5	5.7	5.2	6.5
Service workers	14.1	14.7	15.6	14.1	12.1	14.1	15.2	14.6	14.6	16.2	13.2	16.6	14.9	14.6	17.7
Security workers	0.5	0.0	0.2	0.3	0.6	0.0	0.7	0.4	0.0	0.3	0.4	0.7	1.5	0.0	0.0
Agriculture, forestry and fishery workers	1.0	0.5	1.0	0.7	0.0	0.0	0.0	0.0	0.5	0.0	0.0	0.7	1.2	0.0	2.2
Manufacturing process workers	0.5	5.6	3.8	0.3	1.6	3.5	7.1	0.7	1.0	2.1	0.4	1.4	0.9	0.8	3.6
Transport and machine operation workers	0.0	0.2	0.2	0.3	0.0	0.0	0.0	0.0	0.0	0.3	0.0	1.4	0.3	0.5	0.0
Construction and mining workers	0.0	0.2	0.3	0.0	0.0	0.0	0.0	0.4	0.0	0.0	0.0	0.0	0.0	0.0	0.0

Carrying, cleaning, packaging, and related workers	0.7	1.1	0.5	0.3	2.2	0.3	1.5	0.7	0.5	1.3	0.8	0.7	0.6	1.0	0.0
Homemaker	35.5	29.1	27.8	34.5	36.7	27.6	17.8	31.3	30.2	34.2	44.9	22.8	19.1	39.8	15.9
Others (students, inoccupation, workers not classifiable by occupation)	4.2	5.2	3.7	7.0	4.4	3.2	4.1	4.4	4.2	3.4	4.5	2.8	2.7	4.4	2.5
Smoking habits															
Never smoked	55.9	52.1	57.6	64.5	64.5	62.7	66.3	65.8	67.2	56.7	67.3	68.5	65.2	65.0	61.6
Ex-smokers who quit before pregnancy	26.2	24.4	24.7	24.4	25.3	24.9	23.7	24.1	25.5	25.6	22.0	24.7	21.2	22.8	22.2
Smokers during early pregnancy	17.8	23.5	17.7	11.0	10.2	12.3	10.0	10.1	7.3	17.6	10.6	6.8	13.6	12.2	16.1
Passive smoking (presence of the smokers at home)[a]															
No	82.4	82.9	83.5	92.4	90.3	91.0	91.3	93.5	89.3	82.9	89.0	93.4	84.9	87.3	82.4
Yes	17.6	17.1	16.5	7.6	9.7	9.0	8.7	6.5	10.7	17.1	11.0	6.6	15.1	12.7	17.6
Alcohol consumption															
Never drank	29.7	30.6	31.2	32.1	32.1	38.9	35.2	44.6	32.3	38.6	32.1	34.2	27.1	34.5	35.0
Ex-drinkers who quit before pregnancy	14.1	21.9	18.2	23.7	17.3	19.6	21.9	18.0	18.2	18.9	19.1	25.3	18.0	17.1	16.4
Drinkers during early pregnancy	56.2	47.4	50.6	44.1	50.6	41.5	43.0	37.4	49.5	42.5	48.8	40.4	54.9	48.4	48.6
Body mass index before pregnancy															
<18.5 kg/m^2	14.9	15.0	13.9	13.0	13.0	21.3	18.9	15.8	18.2	16.1	17.5	16.4	15.6	18.1	14.3
18.5–24.9 kg/m^2	76.4	69.6	73.6	77.6	74.7	71.1	74.1	75.5	72.4	73.3	72.0	74.0	73.5	72.5	71.1
≥25 kg/m^2	8.7	15.4	12.5	9.4	12.3	7.6	7.0	8.6	9.4	10.6	10.6	9.6	10.9	9.3	14.6
Parity															
0	46.6	37.4	39.9	43.7	42.6	43.8	45.6	42.4	39.6	38.6	43.1	43.8	41.0	39.9	37.5
1	38.7	39.6	38.6	33.2	39.8	35.0	36.7	40.6	41.7	40.7	41.9	34.9	41.9	37.8	30.4
≥2	14.7	23.0	21.5	23.1	17.6	21.2	17.8	17.0	18.8	20.7	15.0	21.2	17.1	22.3	32.1

SD, standard deviation.

[a] Excluding smokers during early pregnancy

eTable 5. Selected baseline profile of fathers according to Regional Centre in the JECS Sub-Cohort Study

Variables	Hokkaido (%)	Miyagi (%)	Fukushima (%)	Chiba (%)	Kanagawa (%)	Koshin (%)	Toyama (%)	Aichi (%)	Kyoto (%)	Osaka (%)	Hyogo (%)	Tottori (%)	Kochi (%)	Fukuoka (%)	South Kyushu / Okinawa (%)
Number of their partner's pregnancies (Main Study)	2,817	4,143	8,632	3,864	2,418	5,001	3,259	2,547	3,106	2,975	1,868	1,148	2,372	3,784	3,468
Number of their partner's pregnancies (Sub-Cohort Study)	157	197	428	218	114	280	168	140	160	152	99	72	140	202	169
Age when their children were born, years															
Total, mean (SD)	34.0 (5.9)	32.6 (6.3)	33.1 (6.3)	33.9 (5.8)	34.2 (5.9)	34.1 (5.6)	34.7 (5.9)	33.6 (5.1)	34.7 (5.7)	32.9 (7.0)	32.7 (5.2)	33.7 (6.8)	33.6 (6.4)	33.7 (5.8)	33.4 (6.3)
<25	3.2	7.1	7.9	1.4	1.8	3.2	2.4	2.9	0.6	10.5	2.0	6.9	5.7	4.5	4.7
25–29	18.5	24.9	22.2	22.5	22.8	17.1	19.6	19.3	16.9	23.0	30.3	19.4	25.0	19.3	26.6
30–34	34.4	32.5	29.7	35.3	27.2	33.2	27.4	36.4	35.0	31.6	36.4	33.3	21.4	35.6	28.4
≥35	43.9	35.5	40.2	40.8	48.2	46.4	50.6	41.4	47.5	34.9	31.3	40.3	47.9	40.6	40.2
Occupation during their partner's early pregnancy															
Administrative and managerial workers	5.8	3.6	4.0	5.3	2.7	5.8	1.8	2.9	4.5	4.6	7.1	1.4	4.4	4.1	6.0
Professional and engineering workers	34.4	26.9	32.1	35.9	38.1	36.5	31.1	37.1	38.1	26.5	43.4	32.4	33.6	28.4	34.5
Clerical workers	14.3	8.3	9.7	8.1	9.7	7.2	13.2	12.9	9.0	7.3	5.1	7.0	12.4	10.7	7.7
Sales workers	13.0	9.3	7.6	8.1	12.4	7.6	9.6	13.6	14.2	9.3	13.1	7.0	8.8	11.2	10.7
Service workers	13.6	8.8	9.4	9.1	9.7	13.4	6.6	13.6	14.8	12.6	14.1	19.7	16.1	9.1	12.5
Security workers	5.2	5.2	4.7	6.7	6.2	1.1	1.2	3.6	3.9	6.6	2.0	5.6	5.1	6.1	3.6
Agriculture, forestry and fishery workers	2.6	3.1	3.1	0.5	0.0	2.5	1.8	0.0	0.0	0.7	0.0	2.8	3.7	0.5	6.0
Manufacturing process workers	3.9	15.5	17.0	11.5	7.1	16.6	19.8	5.0	7.7	13.3	7.1	11.3	4.4	13.7	7.7

Transport and machine operation workers	3.3	6.2	5.0	4.3	3.5	3.6	5.4	2.9	1.9	6.6	4.0	4.2	4.4	7.6	5.4
Construction and mining workers	1.3	9.3	5.0	6.2	7.1	4.3	7.2	3.6	2.6	9.3	3.0	7.0	5.8	5.6	6.0
Carrying, cleaning, packaging, and related workers	1.3	1.6	0.7	2.4	0.0	1.4	1.2	2.1	2.6	2.0	0.0	0.0	0.7	1.5	0.0
Homemaker	0.0	1.0	0.0	0.0	0.0	0.0	0.0	0.0	0.0	0.0	0.0	1.4	0.0	0.5	0.0
Others (students, inoccupation, workers not classifiable by occupation)	1.3	1.0	1.9	1.9	3.5	0.0	1.2	2.9	0.7	1.3	1.0	0.0	0.7	1.0	0.0
Smoking habits															
Never smoked	29.4	20.6	30.1	34.7	36.0	26.3	39.5	50.4	44.5	27.8	41.8	31.0	33.1	31.5	26.3
Ex-smokers who quit before their partner's pregnancy	33.3	22.2	21.4	31.5	29.8	28.8	26.9	19.4	24.5	29.8	32.7	36.6	18.0	22.5	25.7
Smokers during their partner's early pregnancy	37.3	57.2	48.5	33.8	34.2	45.0	33.5	30.2	31.0	42.4	25.5	32.4	48.9	46.0	47.9
Alcohol consumption															
Never drank	14.9	18.1	19.6	16.4	26.3	22.3	22.2	28.6	26.3	27.8	26.3	22.2	21.6	20.5	13.7
Ex-drinkers	4.5	3.6	5.2	4.7	0.9	2.5	2.4	2.9	3.2	6.6	3.0	2.8	7.2	4.0	0.6
Drinkers	80.5	78.2	75.2	78.9	72.8	75.2	75.4	68.6	70.5	65.6	70.7	75.0	71.2	75.5	85.7
Body mass index before pregnancy															
<18.5 kg/m^2	1.9	3.1	3.1	1.9	3.5	2.9	2.4	4.4	6.5	4.6	3.1	7.0	3.6	6.0	1.8
18.5–24.9 kg/m^2	67.1	63.5	65.4	72.1	70.2	76.7	70.1	70.8	72.9	62.3	76.5	66.2	66.7	64.8	68.5
≥25 kg/m^2	31.0	33.3	31.5	26.0	26.3	20.4	27.5	24.8	20.6	33.1	20.4	26.8	29.7	29.1	29.7

SD, standard deviation.

eTable 6. Selected baseline profile of children according to Regional Centre in the JECS Sub-Cohort Study

Variables	Hokkaido	Miyagi	Fukushima	Chiba	Kanagawa	Koshin	Toyama	Aichi	Kyoto	Osaka	Hyogo	Tottori	Kochi	Fukuoka	South Kyushu / Okinawa
Number of live births (Main Study)	7,904	9,053	12,857	5,942	6,393	7,162	5,392	5,535	3,901	7,838	5,044	3,033	6,883	7,519	5,692
Number of live births (Sub-Cohort Study)	405	447	637	301	324	359	271	278	194	391	250	150	340	387	283
Singleton births, %	99.5	99.6	98.6	98.7	99.1	98.9	99.6	99.3	97.9	96.9	96.8	95.3	99.4	99.0	97.5
Gestational age at birth, weeks															
Total, mean (SD)	39.2 (1.3)	39.3 (1.3)	39.4 (1.4)	39.2 (1.6)	39.3 (1.4)	39.2 (1.7)	39.3 (1.5)	39.5 (1.3)	39.3 (1.6)	39.3 (1.4)	39.4 (1.5)	39.5 (1.3)	39.1 (1.5)	39.5 (1.3)	39.2 (1.7)
Preterm births (<37), %	4.7	3.6	5.3	6.0	4.0	3.6	3.3	4.7	3.6	5.9	4.8	2.0	4.1	3.6	6.4
Term births (37–41), %	95.3	96.4	94.7	94.0	95.4	96.1	96.7	94.6	96.4	93.9	94.8	98.0	95.9	96.1	93.6
Postterm births (≥42), %	0.0	0.0	0.0	0.0	0.6	0.3	0.0	0.7	0.0	0.3	0.4	0.0	0.0	0.3	0.0
Sex															
Male, %	50.4	51.0	52.0	46.8	51.2	51.8	54.2	47.1	54.1	49.1	57.6	46.7	50.3	51.2	49.5
Female, %	49.6	49.0	48.0	53.2	48.8	48.2	45.8	52.9	45.9	50.9	42.4	53.3	49.7	48.8	50.5
Type of delivery															
Vaginal, %	79.8	85.2	82.9	81.3	83.9	80.3	83.8	83.4	80.8	78.8	77.6	76.0	76.5	85.8	75.6
Caesarean, %	20.2	14.8	17.1	18.7	16.1	19.7	16.2	16.6	19.2	21.2	22.4	24.0	23.5	14.2	24.4
Birth weight, g															
Total, mean (SD)	3,056 (392)	3,078 (396)	3,052 (423)	3,022 (419)	3,027 (424)	2,989 (419)	3,051 (430)	3,058 (395)	3,076 (419)	3,009 (427)	3,052 (434)	2,998 (374)	2,980 (375)	3,061 (396)	3,069 (474)
Singleton births															
Total, mean (SD)	3,059 (390)	3,082 (392)	3,063 (412)	3,037 (400)	3,031 (423)	2,992 (420)	3,055 (426)	3,062 (393)	3,095 (387)	3,031 (410)	3,078 (408)	3,028 (353)	2,986 (369)	3,067 (391)	3,086 (463)
Low birth weight (<2,500), %	6.9	6.7	7.5	7.7	7.2	9.9	6.7	6.5	5.8	9.5	6.6	6.3	7.1	4.7	8.0
Birth height, cm															
Total, mean (SD)	48.9	49.5	49.2	48.5	49.2	49.1	48.8	49.7	49.0	48.4	48.9	48.2	48.6	49.5	48.7

	(2.1)	(1.9)	(2.2)	(2.4)	(2.0)	(2.3)	(2.2)	(1.9)	(2.3)	(2.2)	(2.3)	(2.2)	(2.1)	(2.1)	(2.1)
Singleton births, mean (SD)	48.9	49.5	49.2	48.6	49.2	49.1	48.8	49.7	49.1	48.4	49.1	48.3	48.7	49.5	48.8
	(2.1)	(1.9)	(2.1)	(2.3)	(2.0)	(2.3)	(2.2)	(1.8)	(2.1)	(2.1)	(2.1)	(2.1)	(2.0)	(2.0)	(2.1)
Birth head circumference, cm															
Total, mean (SD)	33.5	33.3	33.3	33.1	33.3	33.1	33.2	33.0	33.3	33.2	33.3	33.1	33.2	33.3	33.1
	(1.4)	(1.4)	(1.5)	(1.5)	(1.5)	(1.7)	(1.5)	(1.5)	(1.6)	(1.4)	(1.5)	(1.3)	(1.4)	(1.4)	(1.5)
Singleton births, mean (SD)	33.5	33.3	33.3	33.2	33.3	33.1	33.2	33.0	33.3	33.2	33.3	33.2	33.2	33.3	33.1
	(1.4)	(1.4)	(1.5)	(1.4)	(1.5)	(1.7)	(1.5)	(1.5)	(1.5)	(1.3)	(1.4)	(1.3)	(1.4)	(1.4)	(1.5)
Birth chest circumference, cm															
Total, mean (SD)	31.9	31.8	31.8	31.6	31.9	31.7	31.9	31.9	31.9	31.7	31.9	31.7	31.6	31.9	31.9
	(1.6)	(1.6)	(1.8)	(1.9)	(1.8)	(1.9)	(1.9)	(1.6)	(1.8)	(1.9)	(1.9)	(1.6)	(1.6)	(1.8)	(1.9)
Singleton births, mean (SD)	31.9	31.8	31.9	31.7	32.0	31.7	31.9	31.9	32.0	31.8	32.0	31.8	31.7	32.0	32.0
	(1.6)	(1.6)	(1.7)	(1.8)	(1.8)	(2.0)	(1.9)	(1.6)	(1.7)	(1.8)	(1.8)	(1.6)	(1.6)	(1.7)	(1.8)

SD, standard deviation.
